# Preliminary *In Vitro* Assessment of Stem Cell Compatibility with Cross-Linked Poly(*ε*-caprolactone urethane) Scaffolds Designed through High Internal Phase Emulsions

**DOI:** 10.1155/2015/283796

**Published:** 2015-05-28

**Authors:** Sylvie Changotade, Gabriela Radu Bostan, Anne Consalus, Florence Poirier, Juliette Peltzer, Jean-Jacques Lataillade, Didier Lutomski, Géraldine Rohman

**Affiliations:** ^1^Université Paris 13, Sorbonne Paris Cité, Laboratoire CSPBAT, UMR CNRS 7244, 99 avenue JB Clément, 93430 Villetaneuse, France; ^2^Université Paris 13, Sorbonne Paris Cité, Laboratoire CSPBAT, UMR CNRS 7244, 74 rue Marcel Cachin, 93017 Bobigny, France; ^3^Institut de Recherche Biomédicale des Armées, Unité de Thérapie Cellulaire et Réparation Tissulaire, Site du Centre de Transfusion Sanguine des Armées “Jean Julliard” de Clamart, BP 73 91223 Brétigny-sur-Orge Cedex, France; ^4^Ecole du Val de Grâce, 1 place Alphonse Lavéran, 75005 Paris Cedex, France

## Abstract

By using a high internal phase emulsion process, elastomeric poly(*ε*-caprolactone urethane) (PCLU) scaffolds were designed with pores size ranging from below 150 *μ*m to 1800 *μ*m and a porosity of 86% making them suitable for bone tissue engineering applications. Moreover, the pores appeared to be excellently interconnected, promoting cellularization and future bone ingrowth. This study evaluated the *in vitro* cytotoxicity of the PCLU scaffolds towards human mesenchymal stem cells (hMSCs) through the evaluation of cell viability and metabolic activity during extract test and indirect contact test at the beginning of the scaffold lifetime. Both tests demonstrated that PCLU scaffolds did not induce any cytotoxic response. Finally, direct interaction of hMSCs and PCLU scaffolds showed that PCLU scaffolds were suitable for supporting the hMSCs adhesion and that the cells were well spread over the pore walls. We conclude that PCLU scaffolds may be a good candidate for bone tissue regeneration applications using hMSCs.

## 1. Introduction

Craniomaxillofacial bone defects can occur as a result of congenital defects, diseases, trauma, and injuries [1]. When the defect site does not exceed a critical size, normal healthy bone can spontaneously regenerate; otherwise the use of bone grafting materials is needed [2]. As a consequence, craniomaxillofacial skeleton regeneration represents a major challenge in the global health problem. Autogenous bone graft is the “gold standard” as it possesses many desirable properties, such as osteoconductivity and osteoinductivity, and produces satisfactory results. However, it is associated with postoperative patient morbidity, harvesting difficulties, donor site pain, and poor contouring and is only present in limited quantities [3, 4]. Therefore, synthetic bone substitutes containing the patient's own bone marrow should be good alternative as they may be designed to possess some of the positive properties of autografts [4].

The ultimate goal of bone tissue engineering is to elaborate biomaterials providing appropriate scaffolding conducive to cell adhesion, maintenance of cell function, vascularization, and bone maturation into the construct. Among the wide variety of degradable polymers that have been investigated, polyester urethane-based biomaterials have been increasingly used since they may provide elastomeric bone graft substitute [5–8]. In comparison with “hard” semicrystalline polyester, elastomeric scaffolds are amorphous materials with additional rubberlike elasticity that would allow a convenient fitting of the materials in the bone defect [9]. Moreover, the intimate contact that could be established between the bone and the material surface should suppress shear forces at the interface, therefore enhancing the proliferation of osteogenic cells and promoting bone regeneration [5]. Furthermore, the mechanical properties and final performance of the polyester urethane-based structure may be improved when designing cross-linked networks [10].

The source of the cells is also a crucial parameter in tissue engineering applications. Indeed, cell and tissue response are two fundamental parameters leading to the success of the biomaterial. Osteoblast, embryonic, and adult stem cells have been considered potential sources for cellular components in bone tissue engineering. In particular, human mesenchymal stem cells (hMSCs) are promising candidates for bone regeneration since they are free from ethical concerns, may differentiate along an osteogenic lineage, and possess nonimmunogenic properties [4, 11, 12]. Recently, a proof-of-concept phase I/II feasibility trial demonstrated that therapies combining hMSCs and scaffolds are safe and efficacious in the regeneration of localized craniofacial bone defects and therefore supports expanded studies on the use of hMSCs in bone tissue engineering [13].

In the present study, we hypothesized that elastomeric scaffolds based on cross-linked poly(*ε*-caprolactone urethane) (PLCU) and obtained through the use of high internal phase emulsions could be a good candidate for bone tissue engineering. Indeed, poly(*ε*-caprolactone) (PCL) is a biodegradable aliphatic polyester whose hydrolysis leads to low-concentrated caproic acid that does not cause a significant negative reaction in the surrounding tissue and that is completely metabolized [14]. Elastomeric networks can be designed by cross-linking triol PCL oligomers with aliphatic diisocyanate which leads to polyurethanes that will degrade in nontoxic amine [15]. Finally, the use of high internal phase emulsion (HIPE) process allows the development of polymeric materials with a multiscale and interconnected porosity that is easy to control through the emulsion parameters [16]. When designing a novel biomaterial and prior to* in vivo* investigation, it is necessary to evaluate the cell and tissue response* in vitro*. Thereby, the elastomeric PCLU scaffolds were tested to determine whether they were compatible with hMSCs in terms of toxicity and ability to support stem cell adhesion* in vitro* at the beginning of the scaffold lifetime.

## 2. Materials and Methods

### 2.1. Materials

Triol PCL oligomers (Mn = 1160 g mol^−1^ as determined by ^1^H NMR), hexamethylene diisocyanate (HMDI), Span 80, and dibutyltin dilaurate (DBTDL) were obtained from Sigma-Aldrich. All solvents were purchased from Fisher and used as received. Phosphate buffered saline solution (PBS), Dulbecco's modified Eagle's medium (DMEM), fungizone antimycotic (Fz), penicillin (Pen), streptomycin (Strep), and trypsin-EDTA were supplied by Gibco Life Technologies. MTT (3-(4,5-dimethylthiazol-2-yl)-2,5-diphenyl tetrazolium salt), trypan blue, collagenase, and paraformaldehyde (PFA) were purchased from Sigma-Aldrich. Fetal bovine serum (FBS) was obtained from PAN-Biotech GmbH. Human mesenchymal stem cells (hMSCs) were obtained by plastic adhesion from bone marrow samples collected from hematologically normal patients undergoing routine total hip replacement surgery. All samples were obtained, after informed consent, from donors of the “Department of Orthopedic Surgery, Hôpital d'Instruction des Armées Percy” (Clamart, France).

### 2.2. Scaffold Elaboration

Porous PCLU scaffolds were obtained through the preparation of HIPE. All glassware and the distilled water were autoclaved (20 min, 120°C) in order to synthesize the scaffold in near-sterile conditions. The vertical PTFE stirrer was sterilized for 3 hours in a 70 vol.% ethanol solution. A representative example of scaffold preparation is detailed; triol PCL oligomers (1.3 g) and Span 80 (1.3 g) were placed in a reactor and dissolved in toluene (7 mL). Thereafter, cross-linking agent HMDI (1.04 mL) and DBTDL catalyst (600 *μ*L) were added under stirring at a speed of 300 rpm using a mechanical stirrer. The stirring speed was then increased to 1000 rpm, and 34 mL of distilled water (0.2 *μ*m filtered) was slowly introduced leading to a stable paste-like emulsion. The HIPE was transferred in a mold and heated in an oven at 55°C for 22 hours and annealed at 100°C for 2 hours. Subsequently, the material was unmolded and squeezed on a paper towel to remove the water and the excess of solvent. After 24 hours of air-drying, the scaffold was cut into discs with a thickness of 2 mm and washed as follows: 48 hours in dichloromethane, 6 hours in dichloromethane/hexane 50/50 vol.%, and finally overnight in hexane. The samples were then dried under vacuum.

### 2.3. Scaffold Characterization

The density of the scaffold was determined using gravimetric analysis. The porosity of the scaffold was calculated using the following equation:

(1) $$\begin{array}{c} \text{Porosity}\;\left(\%\right)=1-\frac{\rho_{\text{porous scaffold}}}{\rho_{\text{non-porous network}}}, \end{array}$$

where *ρ*_porous  scaffold_ and *ρ*_non-porous network_ are, respectively, the densities of the porous PCLU material and the nonporous PCLU material prepared in the same conditions compared to the porous scaffold without the addition of water.

The interconnectivity of the porous structure was measured through swelling measurement using hexane which is a nonsolvent of the PCLU matrix. The volumetric hexane absorption ratio (*r*_*v*_) is the ratio between the volume of hexane imbibed and the volume of the pores in the PCLU scaffold and was calculated using the following equation [17]:

(2) rv%=mw−mdρliquid ×ρporous  scaffold×ρnon-porous networkmd×ρnon-porous network−ρporous  scaffold ×100,

where *ρ*_liquid_, *ρ*_porous  scaffold_, and *ρ*_non-porous network_ are, respectively, the densities of the nonsolvating liquid (hexane), the porous PCLU material, and the nonporous PCLU material prepared in the same conditions compared to the porous scaffold without the addition of water. The mass of the wetted scaffold and the mass of the original dry scaffold are *m*_*w*_ and *m*_*d*_, respectively.

Fourier-transformed infrared (FTIR) spectra, recorded in an attenuated total reflectance mode (ATR), were obtained using a PerkinElmer Spectrum Two spectrometer equipped with UATR accessory and controlled by Spectrum software. The spectra were obtained by accumulating 16 scans in the range 500–4000 cm^−1^ with a resolution of 4 cm^−1^. Background scans were acquired without the sample present. Scaffold morphology and energy-dispersive X-ray spectroscopy (EDX) analyses were performed using an environmental scanning electron microscope (ESEM-Hitachi TM3000) operating at an accelerated voltage of 15 kV and equipped with an EDX probe (Hitachi SwiftED3000). The acquisition time for EDX was set to 196.6 s.

### 2.4. Scaffold Conditioning for* In Vitro* Experiments

PCLU scaffolds were immersed in sterile water for 3 hours under a vacuum system and then washed overnight under stirring in sterile water. Thereafter, scaffolds were immersed for 3 hours in 70 vol.% ethanol under a vacuum system and disinfected for 48 hours in 70 vol.% ethanol. Finally, PCLU scaffolds were rinsed three times in sterile PBS and incubated overnight in DMEM/Pen (100 IU/mL)/Strep (100 *μ*g/mL)/Fz (2.5 *μ*g/mL) at 37°C and 5% CO_2_.

### 2.5. *In Vitro* Cytotoxicity

The PCLU scaffold cytotoxicity was evaluated according to the ISO 10993-5 and ISO 10993-12 standards. Two different methods were used: extract test and indirect contact to PCLU scaffolds. All assays were performed in triplicate.

For extract test, extract medium was prepared as follows: Conditioned PCLU scaffolds were incubated at a concentration of 0.1 g/mL in DMEM/Pen (100 IU/mL)/Strep (100 *μ*g/mL)/Fz (2.5 *μ*g/mL) supplemented with FBS (10%) for 24 hours at 37°C and 5% CO_2_. Culture media alone or containing 0.3 vol.% HMDI were incubated in the same conditions and used, respectively, as blank control or cytotoxic control. For the evaluation of cytotoxicity, hMSCs were cultured in DMEM/Pen (100 IU/mL)/Strep (100 *μ*g/mL) supplemented with FBS (10%) for 24 hours at 37°C and 5% CO_2_ and grown to 80% confluency. After 24 hours, the cell culture medium was removed and replaced with the extract medium, the blank control medium, or the cytotoxic control medium. Cells were then incubated for 24 hours at 37°C and 5% CO_2_ prior to MTT assay and trypan blue staining.

For indirect contact test, hMSCs were cultured in DMEM/Pen (100 IU/mL)/Strep (100 *μ*g/mL) supplemented with FBS (10%) for 24 hours at 37°C and 5% CO_2_ and grown to 80% confluency. Thereafter, conditioned PCLU scaffolds were submerged in the culture medium 2 mm above the cell culture monolayer. Cells were then incubated for 24 hours at 37°C and 5% CO_2_ prior to trypan blue staining. Cells were also treated in the same conditions without scaffold to serve as blank control.

For the evaluation of cell metabolic activity, 10 *μ*L of MTT solution at a concentration of 5 mg/mL and 100 *μ*L of DMEM were added to cells and incubated at 37°C for 2 hours. Then MTT solution was gently removed and 100 *μ*L of DMSO was added to each well. Finally, the optical density (OD) of each sample was read by Microplate Reader (BIORAD) at 595 nm. Relative cell metabolic activity was normalized to the mean of the blank control medium. For the evaluation of cell viability, cells were enzymatically detached using trypsin-EDTA and cell suspension was mixed with trypan blue solution 50 vol.%. After 15 min, stained and unstained cells were counted using a Malassez chamber.

### 2.6. *In Vitro* hMSCs-PCLU Scaffold Direct Interactions

After conditioning, PCLU scaffolds were placed in individual wells of a 24-well plate and incubated in DMEM/Pen (100 IU/mL)/Strep (100 *μ*g/mL) supplemented with FBS (10%) for 24 hours at 37°C and 5% CO_2_. Thereafter, the medium was removed and 30 *μ*L of a cell suspension at 5 × 10^3^ cells/*μ*L was seeded on the scaffold and incubated for 3 h at 37°C and 5% CO_2_. Then, the wells were flooded with 1 mL of complete medium and incubated at 37°C and 5% CO_2_ up to 7 days. The medium was changed every 3 days. The scaffolds were tested in triplicate. After 7 days, the scaffolds were carefully rinsed with PBS and cells were detached using a mixture of trypsin (0.025%) and collagenase (0.05%). The number of cells was determined by counting with a Coulter Counter Z1 (Beckman). For the imaging study, scaffolds were fixed in PFA (4%), rinsed, and left in ethanol 70 vol.% up to observation. Images were carried out using a Hitachi TM3000 environmental scanning electron microscopy (ESEM) operating at 15 kV and equipped with a Peltier stage operating at −4°C.

### 2.7. Statistical Analysis

Statistical analysis was performed using the Statistical Package for the Social Sciences (SPSS) software. For every population, group normality was checked by a Kolmogorov-Smirnov's test. The homogeneity of variances between the groups was assessed by a Levene's test. Student's* t-*test or one-way ANOVA was performed for the inspection of statistical differences between the means. Finally, Tukey's and Scheffe's post hoc tests were carried out. In all statistical evaluations, *p* < 0.05 was considered statistically significant.

## 3. Results and Discussion

### 3.1. PCLU Scaffold Elaboration

Porous polymeric scaffolds can be obtained using a HIPE method in which the internal phase volume (dispersed phase) is greater than 74.05 vol.% [18]. After removal of the internal phase, foams with micrometer-to-nanometer scale open-pores are created. The HIPE composition and the process variables influence the properties of the final foam. For instance, the volume and the droplets size of the internal phase dictate, respectively, the foam void volume and pore size. The temperature also affects the droplets size since a temperature increase causes an increase of interfacial tension. Moreover, the viscosity of the continuous phase impacts the stability of the HIPE since a highly viscous continuous phase prevents mixing of the system and lowers the incorporation of the dispersed phase in the emulsion. The addition of solvating porogen in the continuous phase allows the development of scaffolds with a fine porous morphology within the pore walls [18].

**Figure 1:**
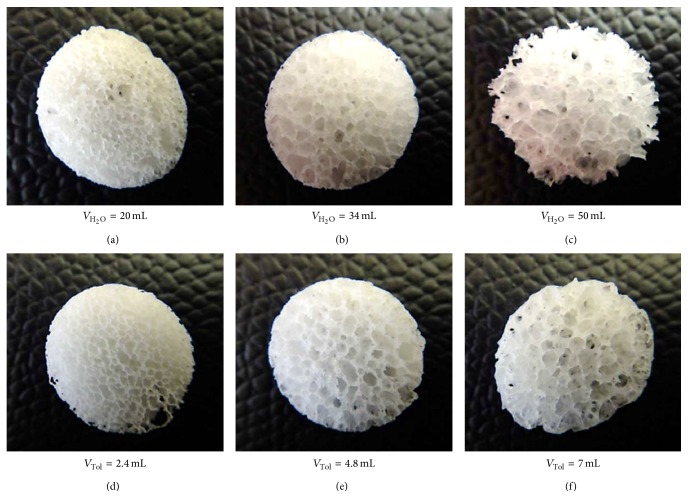
Photos of PCLU scaffolds prepared with different amounts of water or toluene in the HIPE processing (scaffold diameter = 11.5 mm, thickness = 2 mm). (a), (b), and (c): various amounts of water (*V*_H_2_O_) and 4.8 mL of toluene; (d), (e), and (f): various amounts of toluene (*V*_Tol_) with 34 mL of water.

In our study, PCLU scaffolds were synthesized by a one-step polycondensation reaction of HMDI and triol PCL oligomers which were contained in the organic continuous phase of a water-in-oil HIPE. As this process is a one-pot synthesis, it represents a very versatile way of obtaining PCLU scaffolds. Various scaffolds were prepared at 55°C by varying the amount of water in the internal phase (from 69.07 to 84.81 vol.%) or the content of toluene in the continuous phase (from 36.60 to 62.74 vol.%). With these parameters, the HIPE were stable and there was no phase separation during the polycondensation reaction. Higher contents of water led to the collapse of the porous structure under heating, whereas lower content of toluene did not allow obtaining an emulsion due to the viscosity of the continuous phase. The systems described above did not collapse after the removal of the aqueous internal phase and the washing of nonreacted products (surfactant, solvent, catalyst,...). PCLU scaffolds were then produced with a highly porous and interconnected structure. As expected, Figure 1 shows that increasing the amount of water led to an increase of the pore and pore throat size (Figures 1(a), 1(b), and 1(c)). The same trend was observed when increasing the content of toluene (Figures 1(d), 1(e), and 1(f)).

**Figure 2:**
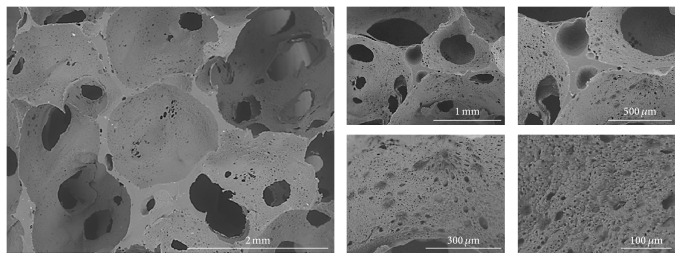
ESEM cross section image of PCLU scaffold prepared with 7 mL of toluene and 34 mL of water.

When developing scaffold for bone tissue engineering, the scaffold macroporosity is necessary for vascularization and new bone ingrowth, whereas the microporosity allows cell attachment and nutrient diffusion. As the human osteon possesses an average size of 223 *μ*m, it is recommended to develop porous materials with the following: a porosity in the range 30–97%, pore size of 100 to 800 *μ*m, and throat pore size above 100 *μ*m [7]. As a consequence, the PCLU selected for the* in vitro* experiments was obtained by using 34 mL of water and 7 mL of toluene (Figure 1(f)). Indeed, this scaffold possesses large pore sizes of 600 to 1800 *μ*m, throat pore sizes as small as 150 *μ*m (Figure 2). Moreover, the use of toluene as solvating porogen in the continuous phase of the HIPE leads to scaffolds with a fine porous morphology within the pore walls (pore size below 150 *μ*m).

### 3.2. PCLU Scaffold Characterization

To look for the possible side reactions happening during the PCLU scaffold elaboration, different types of control materials were synthesized. A control material was prepared in the same conditions compared to the PCLU scaffolds and containing every reactant except the triol PCL oligomers. Stable HIPE were obtained and the paste-like texture was kept during the heating and the annealing processes. However during the washing, the HIPE totally dissolved demonstrating that the surfactant did not participate in the cross-linking reaction. A control material produced only through the reaction of HMDI with water in the presence of DBTDL gave a small brittle white layer over the water surface demonstrating that isocyanate groups may react with water leading to materials containing urea groups.

**Figure 3:**
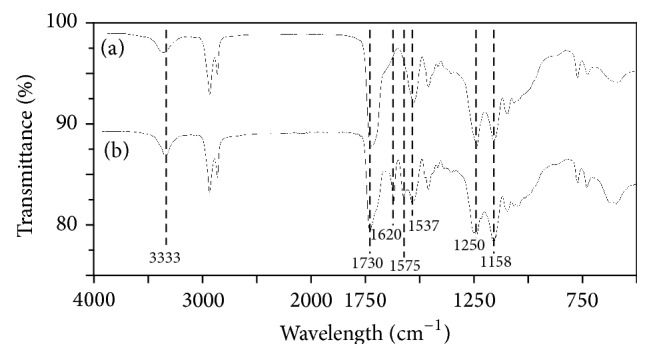
FTIR spectra of (a) nonporous network prepared with 7 mL of toluene without water and (b) PCLU scaffolds prepared with 7 mL of toluene and 34 mL of water.

A nonporous control material was prepared in the same conditions compared to the PCLU scaffolds without the addition of water. The material appeared to be a clear flexible polymer. The FTIR spectrum of the material is given in Figure 3(a). First of all, there is no evidence of isocyanate band at 2260 cm^−1^ indicating the absence of residual isocyanate groups. Secondly the nonporous material exhibits characteristic bands of poly(*ε*-caprolactone urethane) materials. Indeed, it is possible to distinguish [19] urethane hydrogen-bonded –NH stretching at 3333 cm^−1^, asymmetric and symmetric stretching of –CH_2_ groups from PCL and HMDI at 2930 and 2855 cm^−1^, urethane and ester free –C=O groups at 1730 cm^−1^ and hydrogen-bonded –C=O groups for the shoulder of the peak, urethane –NH bending at 1537 cm^−1^, various bending modes of –CH_2_ groups from PCL and HMDI at 1462 and 1357 cm^−1^, –CN stretching and –NH bending associated with aliphatic –R–NH–COO– groups at 1250 cm^−1^, and finally stretching vibrations of the PCL ester group –CO–O–C– at 1158 and 1064 cm^−1^ with the out-of-plane bending of the ester group at 774 cm^−1^. The PCLU scaffold exhibits the same characteristics bands compared to the nonporous control material along with two bands attributed to urea –C=O groups at 1620 cm^−1^ and urea –CNH groups at 1575 cm^−1^ (Figure 3(b)) [20]. These two bands confirmed that water reacts with HMDI since an excess of isocyanate groups was introduced during the HIPE formation. The presence of urea moieties in the PCLU scaffolds acts as hard segments that may increase the scaffold modulus [21].

DBTDL is a common catalyst used in polycondensation reaction and was introduced in the HIPE with an amount of 1.44 wt%. Organotin compounds may be cytotoxic, and it is necessary to check that DBTDL was totally washed out from the scaffold. EDX analyses confirmed the absence of DBTDL in the PCLU scaffold through the absence of tin atom detection (Table 1). It has to be pointed out that the detection of nitrogen in the PCLU structure was not possible due to the X-ray transmission of the detector window which is particularly weak for the N-K line. Taking into account the overlapping of the carbon and nitrogen peaks, the C/O elemental ratio of the PCLU scaffold (2.76) is in good agreement with the theoretical expected value (2.55).

The density of the PCLU scaffold was around 0.14 g/cm^3^ whereas the nonporous control material had a density around 1.03 g/cm^3^. As a consequence, the porosity of the scaffold is around 86%. The value is slightly higher than the one expected by the content of water added to the HIPE (75.3 vol.%). This may be attributed to the formation of urea moieties which is accompanied by the generation of CO_2_ that also acts as porogen agent. Finally, the volumetric absorption of a liquid that did not solvate the PCLU matrix was found to be 100.8 ± 8.0% which indicates that the porous scaffold does not contain closed voids and that the pores are all very well interconnected. This point is of importance as it means that the cells would be able to proliferate in the whole three-dimensional structure, and the vascularization and new bone ingrowth likewise.

### 3.3. *In Vitro* Cytotoxicity and hMSC-PCLU Scaffold Interaction

Cytotoxic effects can prevent the* in vivo* integration of a biomaterial by modifying the natural assimilation process. Therefore, it is necessary to evaluate the biomaterial cytotoxicity through* in vitro* assays according to the ISO 10993-5 and ISO 10993-12 standards. The biomaterial cytotoxicity can result from the original material itself as well as the by-products that may leach out from the material. A few studies have already evaluated biomaterials based on poly(*ε*-caprolactone) and poly(ester urethane) urea and have demonstrated the noncytotoxic nature of the materials when the materials are designed from high molecular weight polymers or from cross-linked oligomers [22–24].

**Figure 4:**
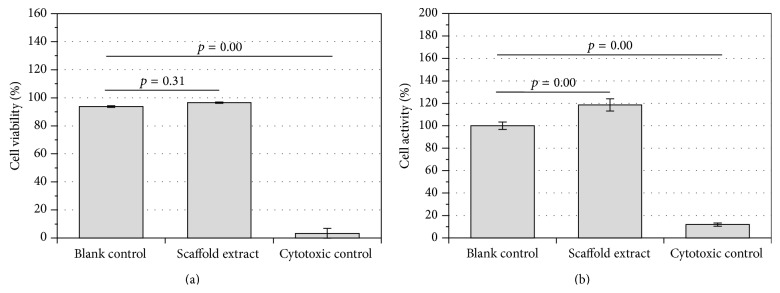
*In vitro* cytotoxicity of the extracts from PCLU scaffold (obtained with 7 mL of toluene and 34 mL of water) compared to blank control (culture medium alone) and cytotoxic control (culture medium with 0.3 vol.% HMDI): (a) hMSCs viability determined by trypan blue assay; (b) hMSCs metabolic activity determined by MTT assay.

The extract test evaluates the cytotoxicity of any leachable products from the material after conditioning and 24 h of incubation. Thereafter, hMSCs cells were cultured in the extract medium for 24 h. As shown in Figure 4(a), the cell viability was statistically significantly higher for the PCLU scaffold extract (%viability = 96.5 ± 0.4%, *p* = 0.00), as well as the blank control (%viability = 93.8 ± 0.6%, *p* = 0.00), compared to the cytotoxic control (%viability = 3.3 ± 3.5%). Moreover, there was no statistically significant difference between the PCLU scaffold extract and the blank control (*p* = 0.31). The cell metabolic activity was also assessed (Figure 4(b)). Again, the cell metabolic activity was statistically significantly higher for the PCLU scaffold extract (*p* = 0.00), as well as the blank control (*p* = 0.00), compared to the cytotoxic control. However, a statistically significant difference between the PCLU scaffold extract and the blank control (*p* = 0.00) with a slight stimulatory effect from the PCLU scaffold extract (%metabolic activity = 118.0 ± 5.4%) was noticed. Overall, as a level of acceptable cell viability and metabolic activity is 70% relative to the blank control, it is possible to conclude that the PCLU scaffold did not induce any release of cytotoxic by-products.

**Figure 5:**
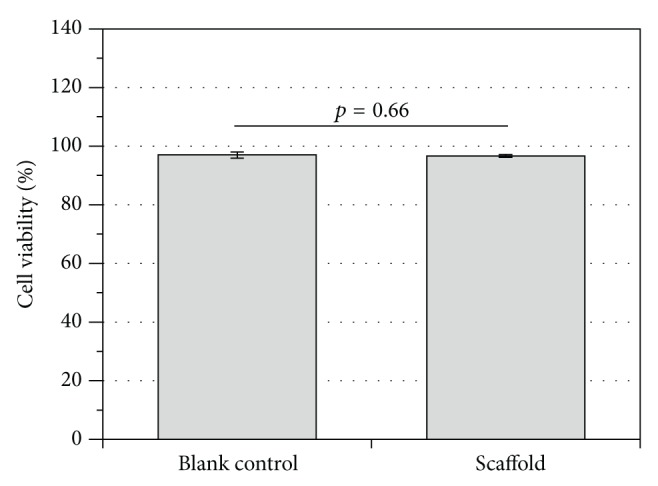
*In vitro* cytotoxicity by indirect contact with PCLU scaffold (obtained with 7 mL of toluene and 34 mL of water) compared to blank control (culture medium alone). hMSCs viability determined by trypan blue assay.

**Figure 6:**
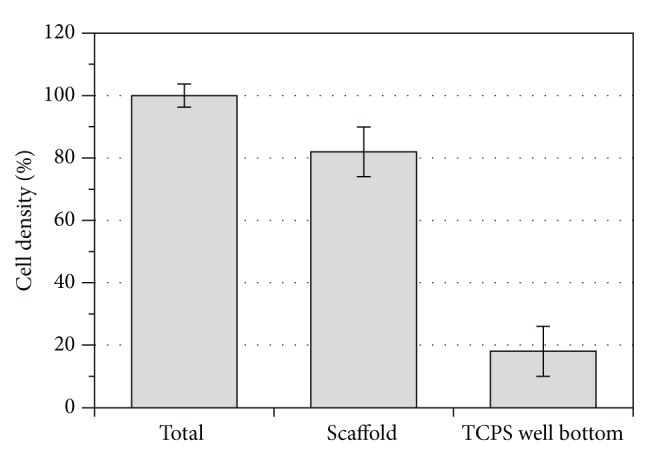
Cell density remained in the PCLU scaffold (obtained with 7 mL of toluene and 34 mL of water) compared to cells adhering to TCPS well bottom and total cell density after 7 days of culture.

**Figure 7:**
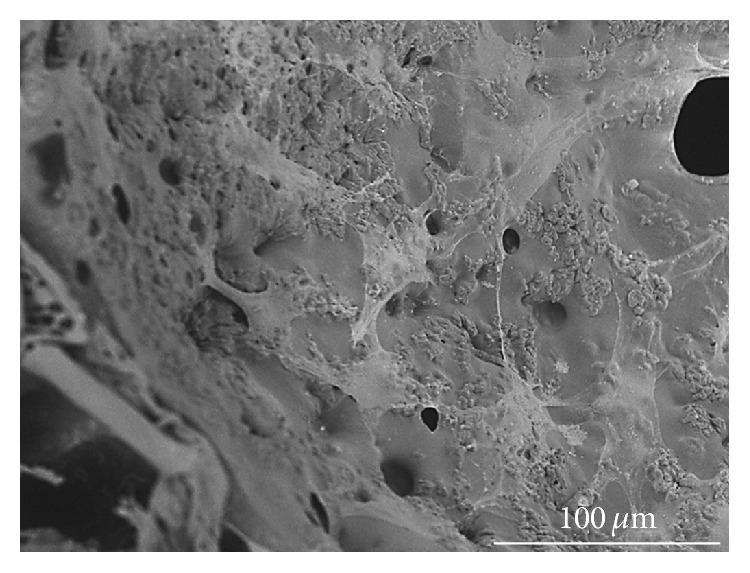
ESEM cross section image of PCLU scaffold (prepared with 7 mL of toluene and 34 mL of water) seeded with hMSCs (7 days of culture and fixed with 4% PFA).

The indirect contact test evaluates the interaction of the material with the cell monolayer without direct physical interaction, and therefore physical disruption of the cell monolayer does not happen during the assay. As shown in Figure 5, there was no statistically significant difference in cell viability when cells indirectly interacted with the PCLU scaffold (*p* = 0.66). Finally, the direct interaction of the hMSCs with the PCLU scaffold was assessed. The incubation time was chosen in order to study the interaction at the beginning of the scaffold lifetime since cell behavior may be affected by various scaffold changes upon long-term degradation.  Figure 6 shows the density of adhered cells 7 days after cell seeding. It appeared that around 82% of the cells still remained in the porous scaffolds whereas 18% fell down onto the TCPS well bottom. This result demonstrates scaffold capability in allowing the adhesion of hMSCs. Moreover, the ESEM analysis of PCLU scaffold cross sections put in evidence that the cells were able to penetrate inside the porous structure (Figure 7). Cells successfully attached to the surface and the pore walls of the porous structure are widely covered with a layer of elongated and well spread cells. Overall, our study demonstrated that PCLU scaffolds appear to not elicit a cytotoxic response and are suitable for supporting the growth of hMSCs.

## 4. Conclusion

The ultimate goal of the study was to produce an elastomeric poly(*ε*-caprolactone urethane) scaffold that could be used in bone tissue engineering and to evaluate the cytotoxicity response of hMSCs exposed to the scaffold or its extracts at the beginning of the cell interaction and the scaffold lifetime. The results presented in this study pointed that PCLU scaffolds are easily obtained through a one-pot HIPE system and that the porous structure and the porosity of the PCLU scaffold may be adequate for cells proliferation, new bone ingrowth, and vascularization. These results also demonstrated that PCLU scaffold exhibited a lack of cytotoxic response and allowed hMSCs adhesion that were elongated over the pore walls. Research is underway to assess scaffold mechanical properties, degradation behavior, and scaffold capabilities in inducing differentiation of hMSCs into an osteoblastic phenotype lineage. Since cell behavior will be affected by various scaffold changes upon long-term degradation, a great attention will focus on the modification of the porous structure and changes in scaffold mechanical properties, as well as variation in the pore wall surface chemistry during long-term* in vitro* and* in vivo* investigations.

## Figures and Tables

**Table 1:** Elemental composition of PCLU scaffold (prepared with 7 mL of toluene and 34 mL of water) as determined by EDX analysis (accelerating voltage = 15 kV, acquisition time = 196.6 s)^a^.

	Element (wt.%)			
	Carbon	Oxygen	Nitrogen	Other
PCLU-theory	65.0	26.9	8.1	—
PCLU scaffold	73.4 ± 3.9	26.6 ± 3.9	0	None

^a^The expected elemental compositions based on the stoichiometry of PCLU are included for comparison. Values are reported as averages and standard deviations. EDX analysis was performed on 8 samples.

## Abbreviations

HIPE: High internal phase emulsion
HMDI: Hexamethylene diisocyanate
hMSCs: Human mesenchymal stem cells
PCL: Poly(*ε*-caprolactone)
PCLU: Poly(*ε*-caprolactone urethane).
